# Virtual reality games for spatial hearing training in children and young people with bilateral cochlear implants: the “Both Ears (BEARS)” approach

**DOI:** 10.3389/fnins.2024.1491954

**Published:** 2024-12-04

**Authors:** Bhavisha J. Parmar, Marina Salorio-Corbetto, Lorenzo Picinali, Merle Mahon, Ruth Nightingale, Sarah Somerset, Helen Cullington, Sandra Driver, Christine Rocca, Dan Jiang, Deborah Vickers

**Affiliations:** ^1^SOUND Lab, Department of Clinical Neurosciences, University of Cambridge, Cambridge, United Kingdom; ^2^Ear Institute, University College London, London, United Kingdom; ^3^Dyson School of Design Engineering, Faculty of Engineering, Imperial College London, London, United Kingdom; ^4^Division of Psychology and Language Sciences, Faculty of Brain Sciences, University College London, London, United Kingdom; ^5^School of Medicine, University of Nottingham, Nottingham, United Kingdom; ^6^Nottingham Hearing Biomedical Research Centre, University of Nottingham, Nottingham, United Kingdom; ^7^Auditory Implant Service University of Southampton, Southampton, United Kingdom; ^8^St Thomas' Hearing Implant Centre, Guy's and St Thomas' NHS Foundation Trust, London, United Kingdom; ^9^Centre for Craniofacial and Regenerative Biology, Faculty of Dentistry, Oral & Craniofacial Sciences, King's College London, London, United Kingdom

**Keywords:** audiology, cochlear implant, spatial hearing, auditory training, sound localization, speech perception, pediatric audiology, deafness (hearing loss)

## Abstract

Spatial hearing relies on the encoding of perceptual sound location cues in space. It is critical for communicating in background noise, and understanding where sounds are coming from (sound localization). Although there are some monoaural spatial hearing cues (i.e., from one ear), most of our spatial hearing skills require binaural hearing (i.e., from two ears). Cochlear implants (CIs) are often the most appropriate rehabilitation for individuals with severe-to-profound hearing loss, with those aged 18 years of age and younger typically receiving bilateral implants (one in each ear). As experience with bilateral hearing increases, individuals tend to improve their spatial hearing skills. Extensive research demonstrates that training can enhance sound localization, speech understanding in noise, and music perception. The BEARS (Both Ears) approach utilizes Virtual Reality (VR) games specifically designed for young people with bilateral CIs to train and improve spatial hearing skills. This paper outlines the BEARS approach by: (i) emphasizing the need for more robust and engaging rehabilitation techniques, (ii) presenting the BEARS logic model that underpins the intervention, and (iii) detailing the assessment tools that will be employed in a clinical trial to evaluate the effectiveness of BEARS in alignment with the logic model.

## Background

Internationally, there are over one million cochlear implant (CI) recipients in the United Kingdom (UK) (Zeng, 2022). Every year, there are ~1,500 new CI recipients in the UK (British Cochlear Implant Group, 2024). Of those who are bilaterally implanted, around 75% are 18 years of age or younger. Extensive evidence supports the conclusion that early cochlear implantation improves speech and language development outcomes in this population (Geers et al., 2003; Sharma et al., 2020; Peixoto et al., 2013), however they often experience significant challenges in speech perception and sound localization, particularly in noisy environments (Zheng et al., 2022; Badajoz-Davila and Buchholz, 2021). Furthermore, bilateral CI users, particularly those sequentially implanted, may experience difficulties in combining sounds from the two implants to create three-dimensional sound (Sparreboom et al., 2012). Some individuals experience “increased effort” when using the second implant due to perceptible differences in sound quality between the devices, which may lead to the rejection of the second implant (Vickers et al., 2021; Myhrum et al., 2017; Watson et al., 2016; Emond et al., 2013). A lack of rehabilitative support to address these challenges has been documented (Mather et al., 2011).

There are currently no standardized clinical fitting protocols, guidance documents, or rehabilitation tools specifically developed to optimize the fitting of bilateral CIs, either in the UK or internationally. Existing rehabilitation techniques with CIs are often unengaging, do not adequately address real-world hearing challenges, and lack targeted training to maximize the benefits of bilateral implantation.

Recognizing the absence of standardized protocols for fitting bilateral CIs, and the need for ecologically valid outcome measures and resources for multi-modal listening training, the BEARS (Both Ears) programme was established. The aim of this paper is to present the BEARS approach and the underpinning logic model, which extends previous research on the development of the BEARS intervention through participatory design methodologies (Vickers et al., 2021).

## BEARS programme logic model

**Figure 1:**
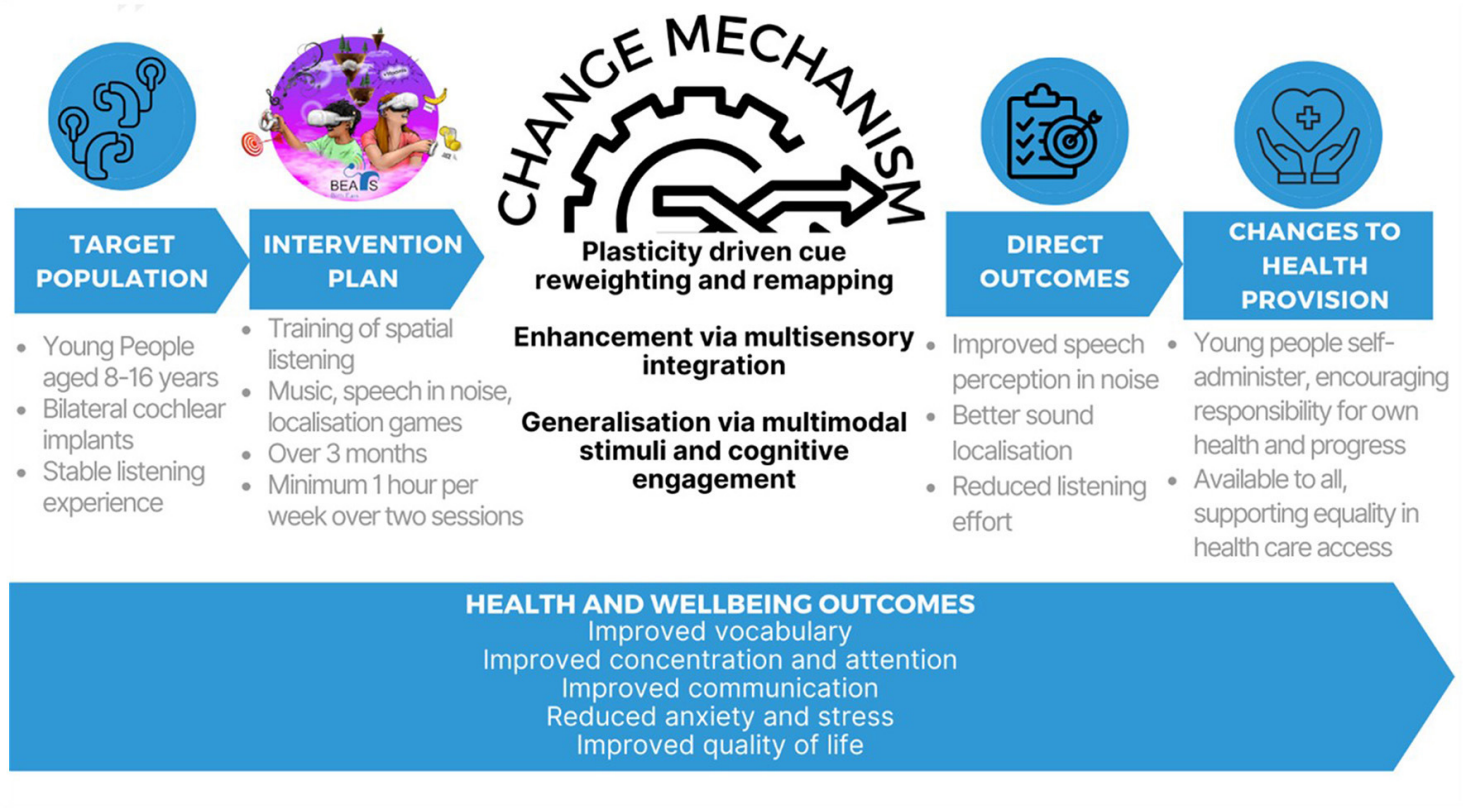
The BEARS logic model created using the Medical Research Council (MRC) framework on complex interventions to improve health (Skivington et al., 2021).

The BEARS programme has involved the development of virtual reality (VR) based spatial hearing games designed to enhance spatial hearing in children and young people (CYP, aged 8–16 years) with bilateral CIs (Vickers et al., 2021). It is informed by a logic model (Figure 1) based on the National Institute for Health and Care Research/Medical Research Council (NIHR/MRC) framework for complex health interventions (Skivington et al., 2021), and has developed both the intervention and outcome measures to rigorously assess intervention effectiveness in a randomized controlled trial (RCT, ISRCTN: 92454702). Logic models are visual representations illustrating the interconnected relationships among various components of a programme or study (Skivington et al., 2021; Funnell and Rogers, 2011).

The BEARS logic model integrates multiple components to assess the intervention and its anticipated outcomes, while also accounting for the specific characteristics of the target patient population. The model outlines the external context for implementation, the mechanisms of change, and the potential effects on healthcare delivery should the intervention demonstrate efficacy. Implementation determinants have also been considered in the development of the BEARS logic model. Moderating and mediating factors include chronological age at first implant, developmental age, training engagement, school setting, duration of hearing before severe-profound deafness, type of intervention device, CI center, number of active CI electrodes, and level of asymmetrical hearing loss. Here, the components of the logic model are presented in more detail.

### Target population

The BEARS logic model is grounded in developmental theory, which accounts for the biological, psychological, social, and emotional changes occurring with age (Piaget, 1971). Within our RCT study population of 384 CYP [power calculation based on pilot data using the BEARS primary outcome measure (spatial speech-in-noise)] with bilateral CIs, it is anticipated that participants will have reached either the “concrete operational” stage, characterized by logical thinking about tangible objects, or the “formal operational” stage, marked by the development of abstract thinking and a more complex understanding of the world. They will also have reached a “cognitive stage” which is linked to the proposed change mechanisms. As hearing abilities improve, this should develop knowledge construction of the world, increasing self-confidence and socio-emotional development (i.e., improve experience, expression, management of emotions and ability to establish positive relationships with others). Participants will be bilateral CI users with a minimum of 6 h of daily usage and stable aided hearing levels (within ±10 dB across 500 Hz−4 kHz), confirmed over at least the two most recent clinical review appointments.

### Intervention plan

Virtual Reality (VR), which relies on immersive, computer-generated audio-visual environments, is increasingly being applied in health research and healthcare delivery. Users interact with VR environments through a head-mounted display and handheld controllers. In auditory research, VR has been utilized to assess listening abilities (Salanger et al., 2020), train localization skills (Shim et al., 2023; Alzaher et al., 2023), and measure the benefits of hearing aids (Grimm et al., 2016). The advantages of VR can include enhanced experimental reproducibility, a reduced need for additional resources and complex speaker-array equipment, as well as increased applicability to real-world scenarios. These benefits may improve the utility of VR based rehabilitation and diagnostic in clinical scenarios. Furthermore, simulating physical spaces through VR, could be advantageous to train, monitor and potentially improve how hearing device users physically respond to sounds e.g., head turn movements and positioning (Grange et al., 2018), in addition to speech perception and sound localization performance.

#### Developing VR games to improve spatial hearing

**Figure 2:**
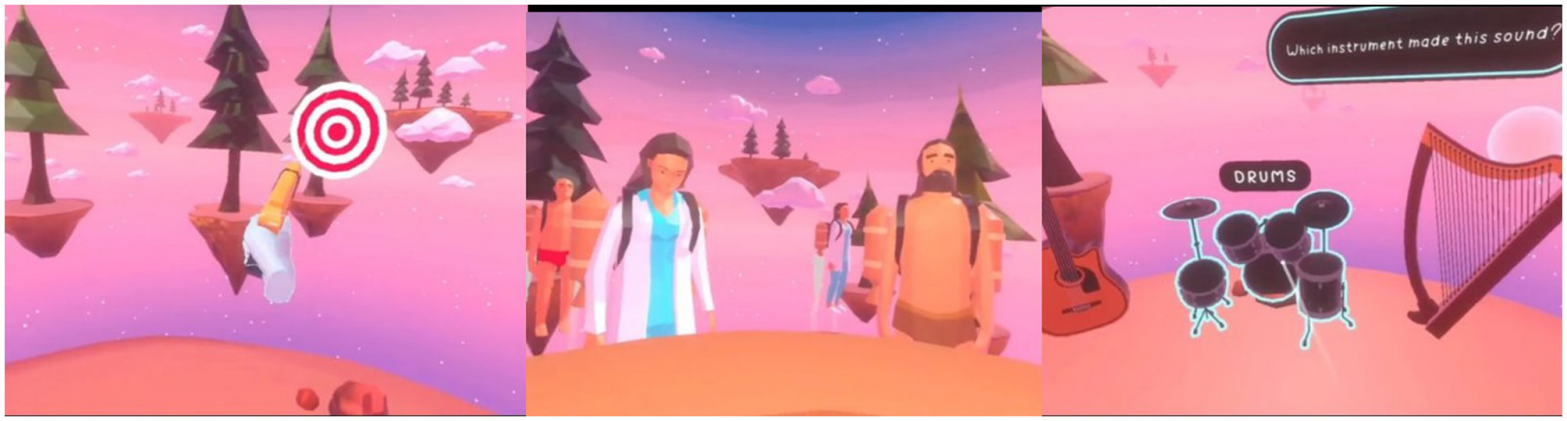
The BEARS games. **(Left)** Sound localization game, where participants identify targets. **(Middle)** Speech-in-noise game where participants follow instructions to serve café customers with food and beverage items. **(Right)** Music game, for participants to complete tasks of pitch discrimination, rhythm repetition, and instrument selection.

The BEARS intervention is a suite of VR games (Figure 2), delivered via the Meta Quest 2 head-mounted device and a pair of headphones, and specifically designed to enhance the spatial hearing abilities of CYP with bilateral CIs. The BEARS intervention was developed using a participatory design approach, as outlined in Vickers et al. (2021), where stakeholders, including CI users, served as co-creators (Vickers et al., 2021). CI users provided valuable feedback on various aspects of the games, such as usability, content, difficulty levels, and settings. Clinicians, including audiologists, speech and language therapists, teachers of the deaf, and music therapists, also played a critical role by evaluating the BEARS training package and suggesting important stimuli for enhancing speech and hearing development. Through collaborative workshops, patients, clinicians, researchers, and engineers reached a consensus that the training package was appropriately designed and ready for use in a randomized controlled trial to evaluate effectiveness.

During this iterative process, modifications were made to ensure the games encompassed a wide range of scenarios, reward systems, lessons and challenges of varying difficulty and clear instructions. They were structured to provide feedback and measure success. Additionally, an iPad version was developed for participants with smaller heads, who find the VR headset uncomfortable, for those who do not like the experience of using the head mounted display, or those with vestibular disorders or significant motion sickness while playing video games. The sound from the VR headset can be presented using inbuilt loudspeakers but due to positioning there was inconsistency in the quality of delivery to the CI processor microphones. There are also options for sound delivery via Bluetooth connection or direct audio-input, but the participatory feedback groups indicated that many individuals were not comfortable in using these listening options and there was greater variability across CI manufacturers. Therefore, headphones were chosen as the most consistent to use for all participants. Various headphones were evaluated for comfort, ease and consistency of use through workshops with the target population, and quality of acoustic representation of the signals and robustness to headphone placement were evaluated by electro-acoustic assessments. AKG k240 headphones were selected for audio delivery with headsets, and with iPads.

The BEARS training package consists of three VR training games to enhance spatial hearing, using target localization, speech perception, and music content (Figure 2). Each game is based on an audio-visual task performed through the VR interface. Players are automatically guided through on-screen visual prompts to support the gameplay with feedback given on their performance. They progress through levels of increasing difficulty. Challenges and lessons are unlocked during the gameplay; the difficulty of the various levels has been calibrated during the participatory design stage and to provide enough content for the whole duration of the trial. The package is designed for self-administration, allowing players the flexibility to engage with the games at any location and time. Workshops with CYP suggested that it is practical to play the BEARS games for at least 1 h per week, divided into a minimum of two 30-min sessions. Clinician workshops recommended that all three games be incorporated into each session to optimize the use of multiple approaches. Informed by these recommendations, relevant literature and device safety guidance (Rechichi et al., 2017; Meta, 2024), no limitations were placed on the number of gaming sessions; however, participants were advised not to exceed 30 min per session. Device datalogging captures detailed gameplay metrics, including session duration, the number of levels unlocked, game points (stars) earned, and the time spent on each game category.

##### Target game

Originally developed to train normal-hearing individuals in sound localization when using non-personalized rendering (Steadman et al., 2019), the target game was later adapted for CI users. In this game, players are initially trained to localize sounds using audiovisual cues. Sounds can originate from any direction around the player, who must identify the target, represented as a bullseye, and direct their controller toward it. At the outset, the bullseye is clearly visible, but as the difficulty levels increase, it gradually disappears, transitioning the task into a purely auditory challenge. Additional challenges involve locating the target signal amidst interfering stimuli or identifying a set of targets in a specific order, further enhancing the training complexity.

##### Speech in noise game

Players are immersed in a virtual café environment, where they are tasked with progressively challenging speech recognition activities. These tasks require players to interact dynamically with the environment by rotating their heads to localize characters who are speaking, and accurately identify the spoken words in the presence of varying levels of background babble. As customers approach from different directions, players must accurately locate them, take their orders, and select the appropriate items from the café counter. The complexity of the game increases with the introduction of background noise and additional interfering tasks within the café setting. A set of advancing levels are also available in a scenario where the player needs to make pizzas, putting the correct ingredients onto the pizzas in the right order and delivering them directly to customers or to delivery staff.

##### Music game

The game aims to enhance perception and localization of musical instruments and lyrics in a range of immersive and interactive soundscapes. Players complete a variety of pitch, timbre, and rhythm discrimination tasks. For example, a pitch discrimination task could involve a participant selecting the location of a pitch-shifted popular song and identifying whether the pitch is higher or lower compared to the original. A rhythm-based task may require participants to use VR controllers to replicate a presented rhythmic beat by playing virtual drums. The music game is based on the Musiclarity web-application, created within the 3D Tune-in project (Reactify, 2024; Cuevas-Rodríguez et al., 2019; Levtov et al., 2016).

### Change mechanisms

Although individuals with bilateral CIs generally exhibit better sound localization and speech-in-noise perception compared to those with a unilateral implant, their performance remains significantly below that of typically hearing children (Sarant et al., 2014; Sparreboom et al., 2015; Lovett et al., 2015; Zheng et al., 2015; Lammers et al., 2014). Extensive research indicates that sound localization can be enhanced through targeted training, with evidence suggesting that plasticity-induced changes can occur in the auditory pathways of both children and adults, facilitated by appropriate training systems (Firszt et al., 2015; Yu et al., 2018; Killan et al., 2019; Mathew et al., 2018). These improvements are underpinned by cue remapping—using new spatial cues to develop a revised localization map—and cue reweighting, which involves emphasizing unaltered cues while disregarding altered ones (Steadman et al., 2019).

Computer-based training offers substantial potential, particularly due to its remote delivery capability and greater engagement. Such training has been shown to improve speech-in-noise perception in CI users (Casserly and Barney, 2017). Research also highlights the efficacy of combined training stimuli. For instance, Cai et al. (2018) found audio-visual training to be more effective than auditory-only training, while Steadman et al. (2019) emphasized the importance of auditory-based interaction during training. A systematic review by Rayes et al. (2019) identified multimodal interventions or a combination of bottom-up and top-down training tasks as the most effective for children with CIs. Whitton et al. (2017) demonstrated that audio-motor perceptual training can improve speech-in-noise intelligibility by up to 25%. Stitt et al. (2019) also illustrated the use of virtual auditory displays to create training environments that teach users to localize sounds using modified localization cues. The inclusion of audio-visual stimuli facilitates task familiarization, while gamification enhances engagement and performance.

It is anticipated that the BEARS intervention, compared to standard care alone, will improve spatial hearing, speech-in-noise perception, and listening ease. These improvements are expected to be driven by plasticity-related processes, training-induced increases in performance change rates and maximum performance, auditory-visual integration, multimodal stimuli, and cognitive engagement-driven generalization. The mechanism of action assumes that the games promote learning.

### Direct outcomes

The evaluation of BEARS follows a mixed methods approach to determine whether BEARS (i) improves speech-in-noise perception in spatial environments, (ii) enhances quality of life, (iii) is cost-effective, and (iv) increases the perceived benefits of everyday listening. A range of tools and measures are utilized to assess outcomes, including some specifically developed as part of the BEARS project. The primary outcome measure is a spatial speech in noise assessment. The Spatial Speech in Noise Virtual Acoustics (SSiN-VA) test simultaneously assesses word identification and relative localization and can provide information about spatial release from masking. It is based on a test initially developed by Bizley et al. (2015) and has been adapted into a virtual implementation (Bizley et al., 2015; Salorio-Corbetto et al., 2022). The virtual adaptive sentence-in-noise task, utilizing the Spatial Adaptive Sentence List (Sp-ASL; MacLeod and Summerfield, 1990), is administered in accordance with the BKB-SIN task protocol (Bench et al., 1979). These virtual outcome measures are carried out with an iPad and calibrated headphones, and were developed in response to the limited availability of multi-speaker arrays for spatial hearing assessments in many audiology departments (Parmar et al., 2022). They are intended to make speech-in-noise testing more accessible and efficient for audiologists, and can be adapted for different populations and clinical purposes.

### Health provision, health, and wellbeing outcomes

A bespoke quality of life measure, the York Binaural Hearing Related Quality of Life—Youth (YBHRQL-Y) has been developed as part of the BEARS programme (Somerset et al., 2023). This measure has been re-operationalized for use with CYP from the original adult YBHRQL developed by Summerfield et al. (2022). Other health economics questionnaires include the Health Utilities Index 3 (HUI-3; Horsman et al., 2003) the Child Health Utility instrument (CHU-9D; Furber and Segal, 2015). The economic evaluation will calculate incremental cost per quality-adjusted life-year (QALY) gained by offering BEARS and usual care compared to usual care, from a National Health Service (NHS), Personal Social Services and Local Education Provider perspective.

A longitudinal qualitative design is being used to explore CYP's experiences of everyday listening, and to contribute to understanding how the BEARS intervention may lead to perceived changes to that experience. Semi-structured online interviews are being carried out with a subset of 40 participants from both BEARS and usual care arms, at baseline and again after 3 months. In addition, all participants in both arms of the trial are asked to respond to open-ended survey questions at successive timepoints throughout the study (baseline, 3 and 12 months). The interview and survey questions have been co-produced in sessions with deaf CYP. Interview and survey data will be analyzed thematically using a Framework approach (Parkinson et al., 2016). Findings will be discussed with deaf CYP to explore whether the trial data resonates and reflects their own lived experiences as users of CIs.

## Conclusion

The BEARS programme comprises a suite of VR games specifically designed to enhance spatial hearing in CYP with bilateral CIs. The development of the BEARS intervention is grounded in evidence presented above, supporting the efficacy of sound localization training, the application of VR technologies, multi-modal training approaches, and the necessity for rehabilitation methods that are both effective and engaging for CYP. These games were co-developed with input from bilateral CI users and other key stakeholders, ensuring their relevance and appeal to the target population. This work is aligned with key objectives outlined in the UK's NHS Long Term Plan (National Health Service, 2019), which emphasizes the importance of expanding digital tools and services to empower patients and support healthcare professionals.

The effectiveness of the intervention will be evaluated within an RCT (ISRCTN: 92454702). The unblinded, multi-center RCT is currently underway to evaluate the effectiveness of a 3-month spatial-listening training programme delivered via the BEARS platform, in addition to usual care, compared to usual care alone. The trial aims to assess improvements in spatial hearing abilities, quality of life, and cost-effectiveness. The study is being conducted across 11 cochlear implant centers in the UK, with a target recruitment of 384 bilateral implanted 8- to 16-year-olds. A 12-month follow up session will assess retention and longer-term effects.

In accordance with the NIHR/MRC framework for complex health interventions (Skivington et al., 2021), we are collaborating with participants, clinicians, and researchers to develop a comprehensive scale-up and implementation strategy. This strategy addresses the immediate challenges of integrating the BEARS intervention into clinical practice, alongside long-term considerations such as ongoing game development, equipment maintenance, and ensuring equitable access. Furthermore, we are partnering with international collaborators to explore the feasibility of global implementation of the BEARS intervention. A critical element of this effort is the BEARS process evaluation, which aims to explore trial compliance, and verify the mechanistic assumptions underlying the intervention's outcomes, and to determine opportunities for optimisation (Moore et al., 2015). Insights from the process evaluation will guide the refinement of the implementation strategy and provide essential information for decision-makers seeking to deploy the intervention across varied settings. To mitigate bias, the process evaluation will be conducted independently of the clinical trial, with data collected by individuals not involved in the design or delivery of the intervention.

The BEARS programme plays a significant role in advancing remote care resources, offering novel interventions that empower patients to take greater ownership of their rehabilitation while potentially alleviating the burden on healthcare providers. For younger populations, the implementation of VR provides a more engaging alternative to traditional auditory rehabilitation methods. The use of participatory design in the development of BEARS games and outcome measures (Vickers et al., 2021) improves their relevance to the target population, thereby enhancing the likelihood of adoption and sustained use.

## Data Availability

The original contributions presented in the study are included in the article/supplementary material, further inquiries can be directed to the corresponding author.
